# Comparative three-dimensional analysis of initial biofilm formation on three orthodontic bracket materials

**DOI:** 10.1186/s13005-015-0062-0

**Published:** 2015-04-10

**Authors:** Marc Philipp Dittmer, Carolina Fuchslocher Hellemann, Sebastian Grade, Wieland Heuer, Meike Stiesch, Rainer Schwestka-Polly, Anton Phillip Demling

**Affiliations:** Center of Dentistry, Oral and Maxillofacial Medicine, Hannover Medical School, Carl-Neuberg-Strasse 1, Hannover, 30625 Germany; Department of Orthodontics, Hannover Medical School, Carl-Neuberg-Strasse 1, Hannover, 30625 Germany; Department of Prosthetic Dentistry and Biomedical Materials Science, Hannover Medical School, Carl-Neuberg-Strasse 1, Hannover, 30625 Germany

**Keywords:** Biofilm, CLSM, Gold, Stainless steel, Ceramic, Orthodontics, Brackets

## Abstract

**Introduction:**

The purpose of the present study was to investigate and compare early biofilm formation on biomaterials, which are being used in contemporary fixed orthodontic treatment.

**Methods:**

This study comprised 10 healthy volunteers (5 females and 5 males) with a mean age of 27.3 +–3.7 years. Three slabs of different orthodontic materials (stainless steel, gold and ceramic) were placed in randomized order on a splint in the mandibular molar region. Splints were inserted intraorally for 48 h. Then the slabs were removed from the splints and the biofilms were stained with a two color fluorescence assay for bacterial viability (LIVE/DEAD BacLight–Bacterial Viability Kit 7012, Invitrogen, Mount Waverley, Australia). The quantitative biofilm formation was analyzed by using confocal laser scanning microscopy (CLSM).

**Results:**

The biofilm coverage was 32.7 ± 37.7% on stainless steel surfaces, 59.5 ± 40.0% on gold surfaces and 56.8 ± 43.6% on ceramic surfaces. Statistical analysis showed significant differences in biofilm coverage between the tested materials (p=0.033). The Wilcoxon test demonstrated significantly lower biofilm coverage on steel compared to gold (p=0.011).

Biofilm height on stainless steel surfaces was 4.0 ± 7.3 μm, on gold surfaces 6.0 ± 6.6 μm and on ceramic 6.5 ± 6.0 μm. The Friedman test revealed no significant differences between the tested materials (p=0.150). Pairwise comparison demonstrated significant differences between stainless steel and gold (p=0.047).

**Conclusion:**

Our results indicate that initial biofilm formation seemed to be less on stainless steel surfaces compared with other traditional materials in a short-term observation. Future studies should examine whether there is a difference in long-term biofilm accumulation between stainless steel, gold and ceramic brackets.

## Introduction

Contemporary fixed orthodontic therapy comprises a variety of biomaterials, which have been introduced in orthodontics since the last century. In the early part of the 20th century, gold was routinely used for many orthodontic appliances like bands, wires and ligatures [1]. Since the 1930s stainless steel was available, but it was not until approximately 1960 that stainless steel was preferred to gold [2]. Thank the increasing esthetical demand of patients, in the 1980s ceramic brackets came into existence [3-5]. In the beginning of the new century, gold was reintroduced in fixed orthodontics due to its use in CAD-CAM design of customized lingual brackets [6-8].

Several clinical studies indicate that the nature of the used biomaterial has a significant impact on biofilm formation in the short-and long-term. Especially the physico-chemical properties of the surfaces are thought to be responsible for an influence on bacterial adherence and accumulation [9-13]. In multiple studies it was found that ceramic materials were covered less by microorganisms than gold, natural dental hard substances and composites [14-18]. Furthermore, studies indicate that metals like gold and amalgam exert an influence against the adhering biofilm by damaging or killing bacteria to a certain extend [14,19].

Despite the antimicrobial potential of orthodontically used biomaterials, the side effects of fixed orthodontic therapy have been described comprehensively in the literature. Insertion of fixed appliances changes the oral microbiota by affecting its quantity, composition, metabolism and pathogenicity [20-23], which results in a higher incidence of gingival inflammation and caries lesions [23-27].

However, iatrogenic side effects of fixed orthodontic treatment might be reduced by the use of biomaterials with a lower biofilm formation. Changes in this variable might facilitate the prevention of caries and gingivitis in the long-term.

Therefore, the objective of the present study was to compare early biofilm formation on biomaterials which are used in contemporary fixed orthodontic treatment.

The null hypothesis of this study was that there would be no statistically significant difference between stainless steel, ceramic and gold in biofilm accumulation after a period of 48 hours.

## Materials and methods

The present study was approved by the Ethics Committee of Hannover Medical School (ethical vote no. 4347) and comprised 5 females and 5 males with a mean age of 27.3 ± 3.7 years. The examination was preformed with the understanding and written consent of each volunteer.

Using nQuery Advisor 5.0 (Statistical Solutions, Saugus, MA, USA), power and sample sizes were calculated. The study was designed to detect a difference of 2.0 ųm in height and 25% in biofilm coverage while assuming a standard deviation of 2.0 ųm in height and 25% in biofilm coverage for the within subject differences. This corresponds to an effect size of 1.0. The sample size to achieve a power of 80% to detect an effect size of 1.0 in a pairwise comparison using the Wilcoxon-test at the level of alpha=0.05 was calculated as n=10.

All volunteers were clinically examined for the exclusion of periodontitis. Recorded parameters were Plaqueindex (PI), Pocket probing depth (PPD) and Bleeding on probing (BOP) [28,29]. Selection of first and third or second and fourth quadrants was performed in randomized order (block randomization with a block size of ten). Only volunteers with PI ≤ 25% and PPD ≤ 4 mm were included in the study. Further criteria for exclusion were systemic illness, pregnancy, removable partial dentures, smoking and antibiotic therapy during the last 6 weeks before the study. Volunteers were advised not to brush their teeth and not to use antimicrobial mouth rinses during the 48 hour period of the present study.

In the present study initial biofilm formation on three traditional biomaterials used in orthodontic treatment was investigated. These were stainless steel (Victory Series, 3 M Unitek, Monrovia, CA, USA), gold (Incognito 3 M Unitek, Monrovia, CA, USA), and ceramic (Clarity, 3 M Unitek, Monrovia, CA, USA). 10 samples per bracket material were obtained from commercially available stock and received a similar surface treatment: the slabs were grounded and polished with grinding paper (grit sizes P600, P1000, P1200, P2400, P4000, Buehler, Düsseldorf, Germany). After polishing all samples, the roughness depths of the different biomaterials were measured at a random area of 90 μm x 90 μm, using an Atomic Force Microscope (AFM) (MFP-3D, Asylum Research, Santa Barbara, CA, USA).

After clinical examination impressions of the lower and upper jaws were taken (Alginoplast, Heraeus Kulzer, Hanau, Germany). On the lower cast a splint was manufactured by use of a viscous hard transparent foil (Erkodur, Erkodent, Pfalzgrafenweiler, Germany; Palapress, Heraeus Kulzer, Hanau, Germany) using a thermoforming technique. The casts were mounted in an articulator (Protar 5, KaVo, Leutkirch, Germany) and the splint was grinded in uniform contact.

The three tested biomaterials (stainless steel, gold and ceramic) were mounted with Tetric Flow (Ivoclar Vivadent, Schaan, Liechtenstein) on the splint, placing these in the mandibular molar region on the buccal site. Afterwards, all specimens were degreased by alcohol. The selection of right and left quadrants was randomized by using a random list. Splints were inserted for 48 hours.

Afterwards splints were removed from the oral cavity and the samples were detached from the splints without destruction of the biofilm and stored in phosphate buffered saline (PBS).

For fluorescence staining of the bacteria an assay for bacterial viability (LIVE/DEAD BacLight–Bacterial Viability Kit 7012, Invitrogen, Mount Waverley, Australia) was used. After staining the samples according to the manufacturer protocol the biofilm formation was analyzed by using a confocal laser scanning microscope (CLSM) (Leica upright-MP Microscope, Leica Microsystems GmbH, Germany).

For analysis of biofilm coverage five randomized areas on each biomaterial were evaluated and from each area a surface picture was obtained in tenfold resolution. Ten pictures vertically to the surface were taken for the evaluation of biofilm thickness at the same area with a 40x resolution and a zoom level of 2.4 set by the software.

The quantitative biofilm surface coverage was calculated using surface-analysis software (Adobe Photoshop; Adobe Systems Inc., San Jose, CA, USA). The bright areas on these pictures represented biofilm coverage, non-covered surfaces appeared dark. Biofilm coverage was calculated concerning these different grey values. Biofilm thickness was measured at five defined coordinates per picture resulting in 250 measuring points for each biomaterial. The mean values and standard deviations of biofilm surface coverage and thickness were calculated for each area of all probes.

Documentation and statistical analysis was performed using the data processing program SPSS/PC-version 20.0 for windows (IBM, Armonk, NY, USA). The Kolmogorov-Smirnov test was applied to test for normal distribution. As data were not distributed normally, data were compared globally using the Friedman test. Pairwise comparison was performed with the Wilcoxon test. All tests were performed two-tailed with a significance level of p=0.05.

## Results

No dropouts were recorded during the study. Periodontal parameters were as follows: PI (Plaque Index) was 23,2 ± 12,9%, PPD (Probing pocket depth) 1,6 ± 0,2 mm and BOP (Bleeding on probing) 3,9 ± 5,4%.

The roughness depths determined by AFM were Ra=0.2 μm on stainless steel, Ra=0.3 μm on ceramic and Ra=0.2 μm on gold.

**Figure 1:**
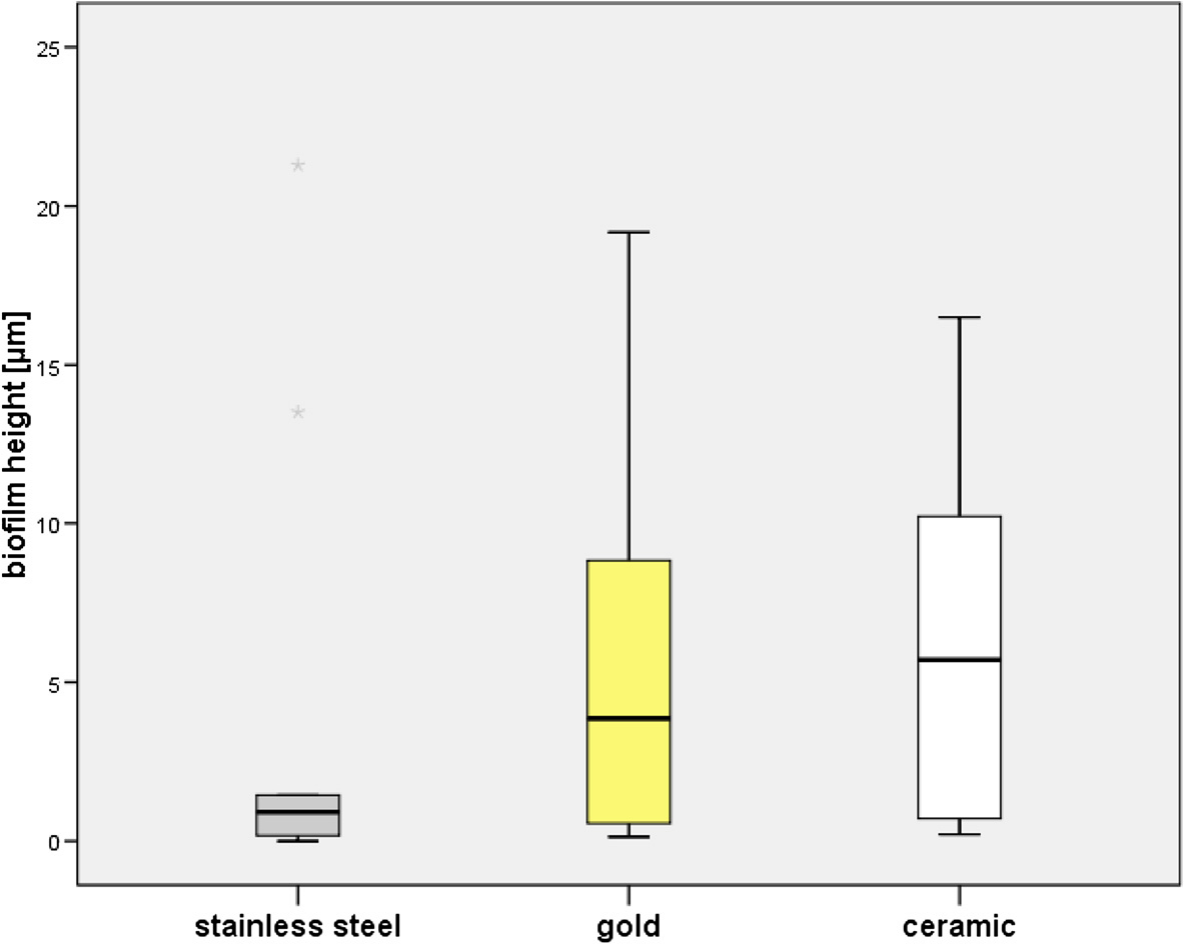
**Boxplot presentation of biofilm height.**

**Figure 2:**
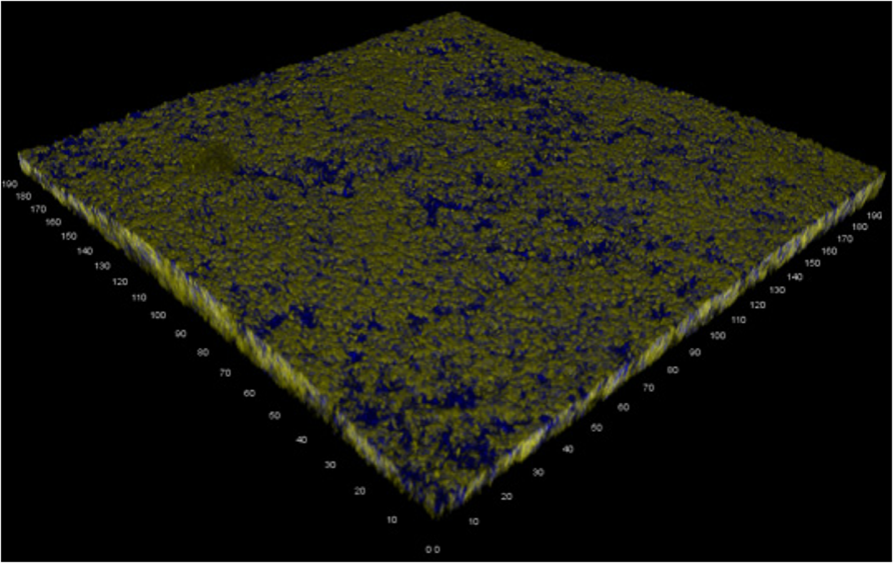
**Three-dimensional reconstruction of biofilm accumulating on stainless steel.**

**Figure 3:**
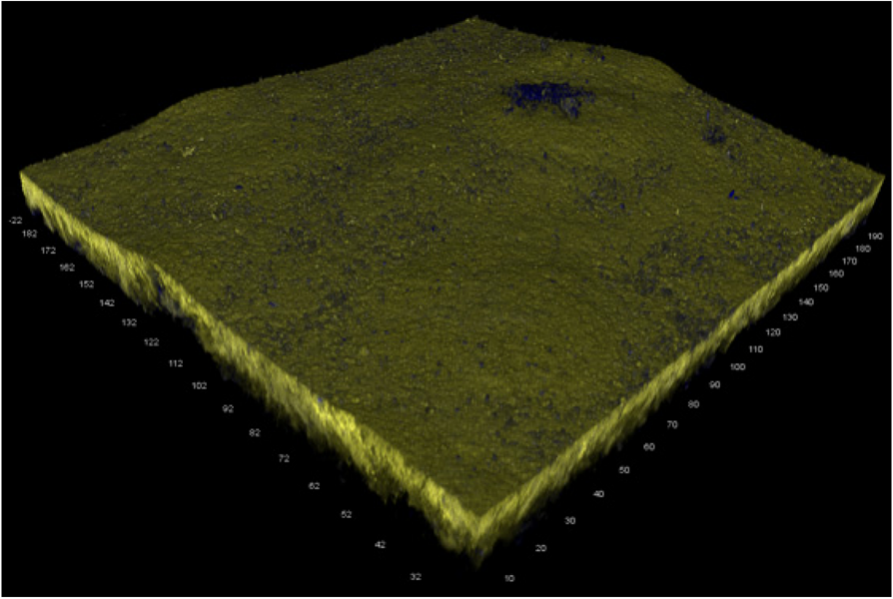
**Three-dimensional reconstruction of biofilm accumulating on gold.**

**Figure 4:**
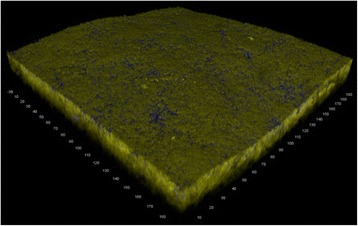
**Three-dimensional reconstruction of biofilm accumulating on ceramic.**

Biofilm was detected by CLSM on all tested bracket materials after exposure to the oral cavity for 48 h. Figure 1 shows the results after analysis of biofilm height with respect to bracket material. On stainless steel surfaces average biofilm height was 4.0 ± 7.3 μm (Figure 2). Biofilm height on gold surfaces was 6.0 ± 6.6 μm (Figure 3), whereas ceramic showed biofilm heights of 6.5 ± 6.0 μm (Figure 4). The Friedman test revealed no significant differences between the tested materials (p=0.150). However, pairwise comparison demonstrated significant differences between stainless steel and gold (p=0.047).

Biofilm covered 32.7 ± 37.7% of stainless steel surfaces, 59.5 ± 40.0% of gold surfaces and 56.8 ± 43.6% of ceramic surface. Statistical analysis showed significant differences in biofilm coverage between the tested materials (p=0.033). The Wilcoxon test demonstrated a significantly lower biofilm coverage on steel compared to gold (p=0.011). Comparison of biofilm coverage between steel and ceramic (p=0.074) and ceramic and gold (p=0.285) showed no significant differences.

## Discussion

Bracket debonding causes an enamel loss of about 50 μm [30] and the mechanical bracket debonding entails the risk of enamel fractures as well [31]. To avoid this enamel damage on permanent teeth and for a secure atraumatic removal, the samples were fixed on a splint. Different kinds of individual splints have been used to collect biofilm in the past [14,32-34]. For the present study, a simple individual removable model was used. Furthermore, accessibility of samples without destruction of fragile initial biofilm was ensured by using the splint model.

The amount of biofilm formation is influenced by the intraoral location [33], whereas posterior regions exhibit a higher plaque formation than anterior ones [35]. This effect is contributed to the self-cleaning mechanisms of the tongue, salivary flow and accessibility to oral hygiene. To avoid mechanical plaque removal by tongue activity in the present study samples were placed bucally in the molar region on the splints. Furthermore, specimens were placed in a randomized order to eliminate the cofounder intraoral localization. In the present study no standardization of diet was applied, which could have influenced the interindividual differences in biofilm formation. Furthermore wearing time of the split was not monitored electronically. As data was only compared intraindividually, this aspect can be neglected in the interpretation of the results.

In vivo studies have shown no differences of the bacterial adhesion and colonization on surfaces with a roughness value ≤ Ra=0.2 μm [36,37]. As the surface roughness of the tested biomaterials was fairly 0.2 μm, the confounder “surface roughness” should not have influenced the results of biofilm thickness and coverage. The reported differences of the biofilm formation between the variety of bracket materials might to some extend be caused by different shapes of brackets. This confounder was avoided by using uniformly shaped test specimens in the present study.

The technique of confocal laser scanning microscopy has been proven to be particularly well suited for the examination of fragile and thin initial microbial biofilms [38-40]. With the aid of CLSM, biofilm formation can be studied in their natural hydrated state, with no requirement of dehydration, chemical fixation or embedding techniques.

The height of biofilm on stainless steel was significant lower than with gold, and almost significant lower compared with ceramic. There is a controversial discussion in the literature which bracket material is more prone to biofilm adherence and plaque retention. To the best of our knowledge, there is a lack of information about in vivo short-term biofilm formation on orthodontic bracket materials. However, by means of mid-and long-term studies data base is inconsistent. A lower initial affinity to bacterial accumulation (Streptococcus mutans) was found in an in vitro study with metal brackets compared to ceramic or plastic brackets [41]. In contrast, no significant differences were found in the accumulation of caries-inducing bacterial species in vivo comparing the plaque-retaining capacity of metal vs. ceramic brackets by counting the levels of different bacterial species on the day of debonding [9]. Nevertheless, other studies indicate a higher plaque-retaining capacity of stainless steel brackets compared to ceramic or plastic brackets due to a higher critical surface tension and total work of adhesion [10]. Furthermore, Escherichia coli, Porphyromonas gingivalis and Streptococcus mutans exhibited a greater affinity to metal brackets compared to ceramics or plastic ones [42,43]. In an in vitro study brackets manufactured from gold were less prone to colonisation of streptococci species [44].

## Conclusion

Comparing findings in literature with data of the present study, there is a significant difference in short-term and mid-or long-term biofilm formation. These differences might be explained by physico-chemical surface alterations that occur over time: Signs of wear caused by food, drink, oral hygiene and corrosion have an influence on surface roughness or surface free energy and would consequently have a significant impact on long-term biofilm formation [11,45,46].

Future studies should examine whether there is a difference in long-term biofilm accumulation between stainless steel, gold and ceramic brackets. Furthermore, the clinical impact on the development of decalcifications and periodontal parameters should be investigated.
